# ESCRT-III and HIV-1 release

**DOI:** 10.1186/1742-4690-10-S1-O3

**Published:** 2013-09-19

**Authors:** Winfried Weissenhorn

**Affiliations:** 1 UVHCI, UMI 3265 University Joseph Fourier-EMBL-CNRS, Grenoble, France

## 

Enveloped viruses such as HIV-1 recruit ESCRT complexes (endosomal sorting complexes required for transport) to escape from cells. Although five complexes constitute the endosomal ESCRT machinery, only ESCRT-III and VPS4 are recruited to all ESCRT-catalyzed processes thereby executing membrane fission. ESCRT-III proteins (CHMP1 to 7, IST1) are cytosolic and transiently recruited to cellular membranes upon activation, which induces their polymerization leading to membrane remodeling. We present cryo electron microscopy data on ESCRT-III CHMP2A-CHMP3 and CHMP2B helical polymer formation and define the basic building block of the ESCRT-III polymer. We show that ESCRT-III proteins become protease resistant and more thermostable upon higher order assembly and discuss structural changes induced by polymerization. An interaction map of ESCRT-II proteins will be presented and discussed with respect to ESCRT-III synergism between CHMP3 and CHMP2A and to a lesser extent with CHMP2B during HIV-1 budding.

